# Inguinoscrotal hernia repair: analysis of the national EVEREG registry

**DOI:** 10.3389/jaws.2026.17525

**Published:** 2026-10-06

**Authors:** M. Verdaguer-Tremolosa, V. Rodrigues-Gonçalves, M. P. Martínez-López, M. López-Cano, in representation of EVEREG group

**Affiliations:** 1 Department of Surgery, UD of Medicine of Vall d’Hebron, Universitat Autònoma de Barcelona, Barcelona, Spain; 2 General and Digestive Surgery Department, Abdominal Wall Surgery Unit, Hospital Universitari Vall d’Hebron, Barcelona, Spain

**Keywords:** groin, hernia, inguinoscrotal, registry, surgery

## Abstract

**Purpose:**

Inguinoscrotal hernia repair has been associated with worse outcomes compared to conventional repairs. Its optimal technique and prognostic factors remain unclear. We aimed to evaluate factors related to complications and recurrence in these repairs based on the EVEREG registry.

**Methods:**

Adult males who underwent elective unilateral inguinoscrotal hernia repair from 2021 to 2024 from the EVEREG registry were included. The primary outcome was postoperative complications. The secondary outcomes were to describe recurrence within 30 days and at latest available follow-up.

**Results:**

A total of 748 patients were included. Most patients (83.4%) underwent open repair, and the preferred mesh position was anterior in 70.8% of cases. Postoperative complications occurred in 6.95% of cases, mostly grade I-II of the Clavien-Dindo classification. Advanced age, diabetes, chronic obstructive pulmonary disease, higher ASA grade, H4-type hernia, open surgical approach, intraoperative complications, and longer operative times were identified as factors associated with postoperative complications (P < 0.001, P = 0.018, P = 0.035, P < 0.001, P = 0.007, P = 0.002, P = 0.039, and P < 0.001). Ambulatory surgery and the involvement of a surgeon with experience in abdominal wall repair were associated with better outcomes (P < 0.001, P = 0.029). Multivariate analysis yielded consistent results regarding protective factors. At maximum follow-up, the presence of a surgeon experienced in abdominal wall repair was the only factor linked to lower recurrence risk.

**Conclusion:**

According to the EVEREG registry, the most common approach for inguinoscrotal hernia repair was open surgery with anterior mesh placement. Postoperative complications occurred mainly in patients with greater comorbidity and hernia complexity and were mostly low-grade. Surgery performed by a surgeon experienced in abdominal wall surgery was associated with fewer postoperative complications, supporting individualised management and consideration of referral for complex cases. The available data do not allow conclusions regarding the superiority of a specific technique or long-term recurrence.

## Introduction

Inguinoscrotal hernias represent a complex type of inguinal hernia characterised by the extension of hernia contents into the scrotum. Their anatomical and technical particularities are associated with increased operative difficulty, a higher risk of complications, and substantial variability in surgical decision-making [1]. Despite these challenges, management strategies for inguinoscrotal hernias continue to rely on individual surgeon experience and limited scientific evidence, particularly under real-world clinical conditions.

Recently, specific recommendations for the management of inguinoscrotal hernias have been published with the aim of reducing recurrence, chronic pain, and postoperative infections [2]. However, the authors acknowledge that these recommendations are mainly based on retrospective series and low-level evidence, limiting their external validity. In addition, they highlight that laparoscopic repair of inguinoscrotal hernias may be technically demanding and associated with relevant conversion rates, emphasising that surgeons managing these cases should be proficient in both open and minimally invasive techniques [2]. Similarly, the updated HerniaSurge international guidelines provide broader recommendations regarding mesh repair, minimally invasive approaches, and prevention of recurrence and chronic postoperative pain [3]. Nevertheless, clinical guidelines do not always fully reflect the heterogeneity encountered in routine surgical practice, where patients present with varying comorbidities, surgical techniques, and follow-up strategies [4]. In this setting, registries offer a valuable source to evaluate outcomes under real-world conditions and assess the applicability of current recommendations across different clinical environments.

The present study analyses data from a national multicentre registry (EVEREG) of patients undergoing unilateral inguinoscrotal hernia repair. The primary aim is to describe the management and short-term outcomes of elective inguinoscrotal hernia repair and explore related factors associated with postoperative complications. Secondary outcomes are to describe recurrence during available follow-up and examine current surgical practice in relation to contemporary recommendations for scrotal hernia management.

## Materials and methods

Including data from more than 200 hospitals, the EVEREG database (http://www.evereg.es) is the national online hernia registry in Spain, whose characteristics have been described elsewhere [5]. The registry prospectively collects anonymised patient data and is maintained by surgeons with an interest in abdominal wall surgery (AWS). It is an audited register that gathers information on patient and hernia characteristics, surgical technical aspects, postoperative complications, and follow-up [6]. Participation in EVEREG is voluntary, and participating investigators commit to the consecutive registration of eligible cases. Therefore, the registry cannot be assumed to capture all inguinal hernia repairs performed in Spain.

Adult patients (≥18 years old) who underwent elective unilateral inguinoscrotal hernia repair between January 2021 and December 2024, as recorded in the EVEREG registry, were included. Scrotal hernia is defined as an inguinal hernia that has descended into the scrotum and causes any degree of scrotal distortion, according to current guidelines [2]. According to the registry, inguinoscrotal hernias were recorded in EVEREG as H3 or H4 according to the Kingsnorth classification: H3 corresponds to an inguinoscrotal hernia reducible with manual manipulation and H4 to an irreducible inguinoscrotal hernia [7]. Cases involving other types of groin hernia repairs or emergency surgeries were excluded. The final data extraction was performed in December 2025, allowing at least 1 year of potential follow-up for patients operated on through December 2024.

Patient demographic data (age, smoking status, body mass index (BMI)), comorbidities (diabetes status, hypertension (HTA), chronic obstructive pulmonary disease (COPD), liver disease, kidney disease, and other relevant medical conditions), and American Society of Anaesthesiologist (ASA) grade [8] were collected. Hernia characteristics, including if it was symptomatic, time since onset of hernia, loss of domain, recurrent hernia, and Kingsnorth hernia classification (type H3 and H4), were registered, as well as wound contamination according to the US Centres for Disease Control and Prevention (CDC) [9, 10]. Specifically, in EVEREG, the loss-of-domain field is conditionally displayed when the hernia is recorded as non-reducible, and the Tanaka index itself is not a mandatory registry field.

Abdominal wall prehabilitation with botulinum toxin and progressive preoperative pneumoperitoneum (PPP) was recorded. Data on surgeries (including ambulatory setting, antibiotic prophylaxis, type of anaesthesia, presence of a surgeon experienced in AWS, surgical approach, surgical technique, and intraoperative complications) were collected. Information about postoperative complications, Clavien-Dindo classification [11], surgical site infection (SSI), surgical site occurrences (SSO; including seroma, hematoma, SSI, and mesh infection), recurrence, length of follow-up, and hospital stay was collected.

This cohort study was conducted in accordance with the RECORD and STROBE statements [12, 13]. The primary outcome was the presence of postoperative complications. Secondary outcomes included recurrence at 30 days post-surgery and recurrence at latest available follow-up.

### Statistical analysis

A descriptive analysis of the sample was initially performed. Categorical variables were summarised as frequencies and percentages, whereas continuous variables were presented as mean ± standard deviation for normally distributed data or as median with interquartile range (25th–75th percentiles) otherwise. Missing data were not imputed. Descriptive and bivariate analyses were performed using the available data for each variable, and denominators are reported whenever they differ from the total cohort. Bivariate analyses were conducted to assess associations between all variables and the occurrence of postoperative complications according to the Clavien-Dindo scale, using the Chi-square test, Fisher’s exact test, Student’s t-test, or Mann-Whitney U test, as appropriate.

Multivariable binary logistic regression was performed to identify factors independently associated with 30-day postoperative complications. Variables with a p value < 0.10 in the bivariate analyses were initially considered for inclusion in each model. Variables that did not retain statistical significance were subsequently removed in a stepwise manner while considering the extent of missing data, potential confounding, possible interactions, and the clinical relevance of each variable based on investigator judgment. Adjusted odds ratios (OR) with 95% confidence intervals (CI) and P values derived from the Wald test were reported. A sensitivity analysis was performed to assess the robustness of the findings. Model discrimination was assessed using the receiver operating characteristic (ROC) curve and the area under the curve (AUC), whereas model calibration was evaluated using the Hosmer–Lemeshow goodness-of-fit test. A two-sided p value < 0.05 was considered statistically significant in all analyses. The statistical software used for the analysis was SPSS 26.0 (IBM Corp.).

## Results

From January 2021 to December 2024, 748 male patients undergoing elective unilateral inguinoscrotal hernia repair were included. The extent of missingness varied between variables and was particularly relevant for some registry fields. Mean age was 66.31 years (SD 14.24) and mean BMI was 27.46 (SD 8.38). According to available data, 20.4% of patients were active smokers (N = 152/743), 138 had diabetes (18.4%), 351 suffered HTA (46.9%), 123 had COPD (16.4%), 25 had liver disease (3.3%) and 44 had adrenal disease (5.9%). Other comorbidities were reported in 20.5% of patients (N = 153). Overall, 28.18% of patients (N = 177/628) were classified as ASA III-V. Most inguinoscrotal hernias were symptomatic at presentation (98%, N = 687/701), and 44.3% of patients (N = 321/725) reported a hernia duration of less than 1 year. According to the Kingsnorth classification, most hernias were classified as H3-type (78.3%, N = 586). Among the 162 irreducible hernias (H4-type), information on loss of domain was available in 108 (66.7%), of which 17 (15.7%) were recorded as loss of domain. Additionally, 11.5% of hernias (N = 86/746) represented recurrences after previous repair.

PPP was performed in 0.8% of cases (N = 6/741) and three patients received botulinum toxin (0.4%). Ambulatory surgery was carried out in 51.3% of cases (N = 384/740), and most of them were type H3 hernias (84.6%, N = 325). In 56.8% of cases, the repair was performed by a surgeon with experience in AWS (N = 420/740). Most of the procedures were conducted under general anaesthesia (63.6%,N = 466/733) and 36.4% under locoregional anaesthesia; 83.2% of patients (N = 616/740) received antibiotic prophylaxis.

Most patients (83.4%, N = 605/725) underwent open repair, and 16.6% received minimally invasive surgery. A total of seven cases (5.5%) required conversion to open surgery. The preferred mesh position was anterior in 70.8% of cases (N = 505/713) and posterior in 29.2% (N = 208/713). Permanent synthetic mesh was used in 99.4% of cases (N = 699/703), while only four cases involved an absorbable synthetic mesh. Among permanent synthetic meshes, polypropylene was the most frequently used material (99.4%). Mesh fixation methods were recorded in 87.43% of patients (N = 657/748; N = 548 for the open approach and N = 109 for the laparoscopic approach). In the open group, mesh fixation methods included absorbable suture in 61.1% of cases (N = 335/548), non-absorbable sutures in 30.1%, tackers in 1.3%, and glue in 1.5%, whereas in 6% of cases no mesh fixation was applied. In the laparoscopic group, the most frequently used mesh fixation methods were tackers in 43.1% of cases (N = 43/109), followed by glue (38.5%), absorbable sutures (8.3%), and non-absorbable suture (0.9%), while no mesh fixation was used in 9.2% of cases.

In 9.6% of cases (N = 38/397), an associated procedure was required, including intestinal resection (N = 2), orchiectomy (N = 1) and other hernia repairs (N = 26), among others. Intraoperative complications occurred in 18/693 cases (2.6%). According to the CDC classification, surgical field was classified as clean or clean-contaminated in 99.9% of cases (N = 739/740 (clean N = 710, clean-contaminated N = 29)) and contaminated or dirty in one case. The mean surgical time was 63.07 min (SD 27.19). The mean length of hospital stay was 5.28 days (SD 75.85).

### Postoperative complications

Postoperative complications were found in 52/748 patients (6.95%). Based on the available data, complications within the first 30 postoperative days were registered as Clavien-Dindo grade I-II in 84.61% of cases (N = 44/52) and grade III-V in 15.38% (N = 8/52). Specific postoperative events, including SSO, were recorded separately in the registry, and more than one event could occur in the same patient. Therefore, among patients with complications and available information for each event, hematoma was recorded in 57/69 patients, seroma in 22/45, SSI in 6/46, and SSO in 58/70. No mesh infections were reported. A recurrence rate of 1.63% was reported during the 30 days after surgery (N = 7/428).

According to data at the latest available follow-up, seroma was reported in 16.1% of patients (N = 5/31), hematoma in 37.9% (N = 11/29), SSI in 3.4% (N = 1/29), and recurrence in 7.5% (N = 3/40). The mean follow-up was 3.36 months (SD 5.88, range 0.5–48 months).

The analysis of factors associated with postoperative complications is presented in Table 1. Advanced age, diabetes, COPD, and higher ASA grade were significantly associated with complications (P < 0.001, P = 0.018, P = 0.035, P < 0.001), as was the H4 type of the Kingsnorth classification (P = 0.007).

**TABLE 1:** Analysis of postoperative complications.

Variable	No complications (N = 696)	Complications (N = 52)	*P*
Age (years) [mean, (SD)]	65.8 (14.3)	72.8 (12.1)	<0.001
BMI (kg/m^2^) [mean, SD]	27.45 (8.64)	27.61 (3.41)	0.154
Diabetes, n/N (%)	122/696 (17.5)	16/52 (30.8)	0.018
COPD, n/N (%)	109/696 (15.7)	14/52 (26.9)	0.035
Current smoking, n/N (%)	142/691 (20.5)	10/52 (19.2)	1
Hypertension, n/N (%)	323/696 (46.4)	28/52 (53.8)	0.3
Liver disease, n/N (%)	23/696 (3.3)	2/52 (3.8)	0.69
Renal disease, n/N (%)	40/695 (5.8)	4/52 (7.7)	0.538
ASA score n/N (%) I-II III/IV	431/585 (73.7) 154/585 (26.3)	20/43 (46.5) 23/43 (53.5)	<0.001
Awareness of the hernia n/N (%) <1 year ≥1 year	297/673 (44.1) 376/673 (55.9)	24/52 (46.2) 28/52 (53.8)	0.777
Symptomatic hernia n/N (%)	636/649 (98.0)	51/52 (98.1)	1
Recurrent hernia n/N (%)	77/694 (11.1)	9/52 (17.3)	0.176
Kingsnorth classification n/N (%) H3 H4	553/696 (79.5) 143/696 (20.5)	33/52 (63.5) 19/52 (36.5)	0.007
Loss of domain n/N (%)	13/94 (13.8)	4/14 (28.6)	0.229
PPP n/N (%)	5/689 (0.7)	1/52 (1.9)	0.355
Botulinum toxin n/N (%)	2/689 (0.3)	1/52 (1.9)	0.196
Ambulatory surgery n/N (%)	373/688 (54.2)	11/52 (21.2)	<0.001
Anaesthesia n/N (%) Regional General	248/681 (36.4) 433/681 (63.6)	19/52 (36.5) 33/52 (63.5)	0.986
Antibiotic prophylaxis n/N (%)	571/688 (83.0)	45/52 (86.5)	0.509
Approach n/N (%) Open Laparoscopy	557/676 (82.4) 119/676 (17.6)	48/49 (98.0) 1/49 (2.0)	0.002
Conversion (n/N, (%))	7/126 (5.56)	0/1 (0.0)	1
Abdominal-wall-experienced surgeon n/N (%)	398/688 (57.8)	22/52 (42.3)	0.029
CDC classification n/N (%) I-II III-IV	687/688 (99.9) 1/688 (0.1)	52/52 (100.0) 0/52 (0.0)	1
Mesh type n/N (%) Synthetic permanent Synthetic absorbable	647/651 (99.4) 4/651 (0.6)	52/52 (100.0) 0/52 (0.0)	1
Mesh position n/N (%) Anterior Posterior	466/665 (70.1) 199/665 (29.9)	39/48 (81.3) 9/48 (18.8)	0.1
Intraoperative complications n/N (%)	14/641 (2.2)	4/52 (7.7)	0.039
Operative time (min) [median, (P25-P75)]	60 (45–72.5)	73 (60–95)	<0.001
Length of hospital stay (days) [median, (P25-P75)]	1 (1–1)	2 (1–4)	<0.001
Recurrence at 30 days postoperative, n/N (%)	3/387 (0.8)	4/41 (9.8)	0.002
Recurrence at the latest available follow-up, n/N (%)	2 (66.7)	1 (33.3)	0.394
Follow-up (months) [mean, (SD)]	3.21 (5.77)	4.88 (7.1)	0.014

BMI, body mass index; SD, standard deviation; P25-P75, percentile 25 – percentile 75; N, number of patients with available information; Min, minutes; COPD, chronic obstructive pulmonary disease; ASA, American Society of Anaesthesiologists; PPP, preoperative progressive pneumoperitoneum; CDC, US Centres for Disease Control and Prevention classification.

The presence of other comorbidities was not associated with significant differences between groups (P = 0.627). Ambulatory surgery and the involvement of a surgeon with experience in AWS were associated with a lower risk of complications (P < 0.001, P = 0.002). An open surgical approach was associated with a higher complication rate (P = 0.002). In contrast, CDC classification was not associated with an increased complication rate; however, the registered number of patients in this category was limited. Similarly, conversion rate was not related to this outcome but available information on this variable was scarce. Mesh position did not appear to influence the complications rate, although anterior repair predominated, and neither did the performance of an additional procedure (P = 1). Intraoperative complications influenced this outcome, although they were recorded in only 18 cases (P = 0.039).

Longer surgical time and hospital stay were both significantly associated with complications (P < 0.001, P < 0.001). Follow-up duration was significantly longer among patients who developed complications (P = 0.014), as was the recurrence rate at 30 days postoperatively (P = 0.002).

In the multivariate analysis (Table 2), ambulatory surgery (OR 0.359, 95%CI 0.168–0.878; P = 0.008) and surgery performed by an AWS-experienced surgeon (OR 0.49, 95%CI 0.259–0.928; P = 0.028) were associated with lower odds of postoperative complications. Similar results were found regarding laparoscopic approach despite scarce data (OR 0.127, 95%CI 0.017–0.962; P = 0.046). Longer operative time remained associated with increased odds of postoperative complications (OR 1.014, 95%CI 1.004–1.024; P = 0.006). Sensitivity analysis yielded consistent results with similar effect estimates and the main significant associations remaining invariable. The model showed an AUC = 0.797 (95% CI 0.732–0.862) for discriminating between patients with and without 30-day postoperative complications (Figure 1).

**TABLE 2:** Logistic regression regarding 30-day postoperative complications.

Variable	Adjusted OR (95% CI)	P
Age (per year)	1.024 (0.996–1.052)	0.092
Diabetes mellitus	1.438 (0.728–2.839)	0.295
COPD	1.68 (0.802–3.516)	0.169
ASA III–IV vs. I–II	2.07 (0.97–4.41)	0.060
Recurrent hernia vs. primary	0.757 (0.265–2.16)	0.603
Kingsnorth H4 vs. H3	1.811 (0.93–3.527)	0.081
Ambulatory surgery	0.359 (0.168–0.768)	0.008
Abdominal wall surgery experienced surgeon	0.49 (0.259–0.928)	0.028
Laparoscopic vs. open approach	0.127 (0.017–0.962)	0.046
Intraoperative complications	2.087 (0.477–9.137)	0.329
Operative time (min)	1.014 (1.004–1.024)	0.006

COPD, chronic obstructive pulmonary disease; ASA, American Society of Anaesthesiologists.

**FIGURE 1:**
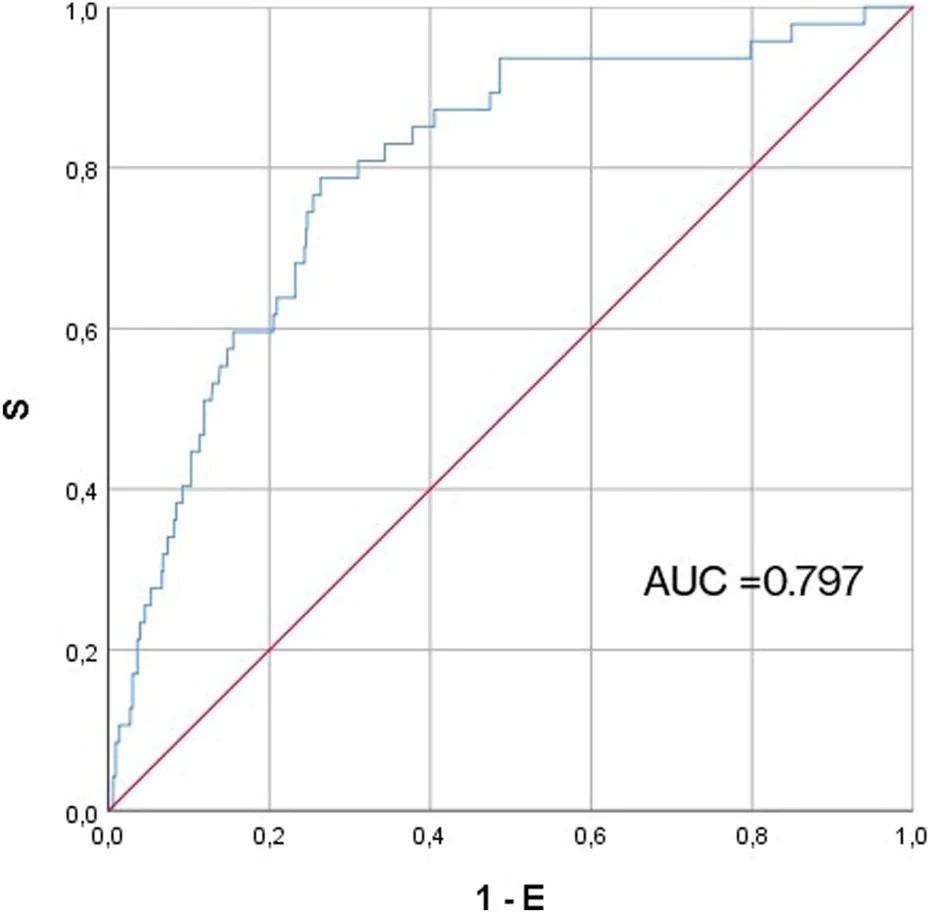
ROC of the multivariate model for 30-day postoperative complications.

### Recurrence

Recurrence at 30 days postoperatively was detected in seven patients. None of the demographic variables were significantly associated with an increased risk of recurrence, nor were the Kingsnorth hernia classification (p = 0.616) or CDC grade (P = 1) (Table 3). Surgical characteristics of the repair did not influence this outcome and neither did the involvement of an AWS-experienced surgeon (P = 1). Similarly, patients with first-month recurrence also had a longer hospital stay (3.5 ± 3.32 vs. 1.41 ± 2.22 days, P = 0.007) and follow-up (4.1 ± 6.1 vs. 0.9 ± 22 months, P = 0.036).

**TABLE 3:** Analysis of recurrence at 30 postoperative days and at latest available follow-up.

Variable	Recurrence 30 POD		P	Recurrence at latest available follow-up		P
	No (n = 421)	Yes (n = 7)	​	No (n = 37)	Yes (n = 3)	​
Age (years), mean (SD)	65.87 (14.43)	73.7 (8.22)	0.157	66.1 (13.55)	58.67 (26.03)	0.692
BMI (kg/m^2^), mean (SD)	27.4 (5.18)	26.16 (3.93)	0.674	27.35 (3.69)	30.7 (4.69)	0.182
Diabetes, n/N (%)	75/421 (17.8)	2/7 (28.6)	0.614	8/37 (21.6)	0/3	1
COPD, n/N (%)	62/421 (14.7)	2/7 (28.6)	0.282	2/37 (5.4)	1/3 (33.3)	0.214
Current smoking, n/N (%)	88/421 (20.9)	1/7 (14.3)	1	6/37 (16.2)	1/3 (33.3)	0.448
Hypertension, n/N (%)	197/421 (46.8)	4/7 (57.1)	0.711	18/37 (48.6)	0/3	0.238
Liver disease, n/N (%)	9/421 (2.1)	0/7	1	1/37 (2.7)	1/3 (33.3)	0.146
Renal disease, n/N (%)	16/421 (3.8)	0/7	1	2/37 (5.4)	0/3	1
ASA score n/N (%) I-II III/IV	297/404 (73.5) 107/404 (26.5)	3/6 (50) 3/6 (50)	0.198	22/36 (61.1) 14/36 (38.9)	1/3 (33.3) 2/3 (66.7)	0.557
Awareness of the hernia n/N (%) <1 year ≥1 year	201/421 (47.7) 220/421 (52.3)	3/7 (42.9) 4/7 (57.1)	1	12/37 (32.4) 25/37 (67.6)	1/3 (33.3) 2/3 (66.7)	1
Symptomatic hernia n/N (%)	414/421 (98.3)	7/7 (100)	1	36/37 (97.3)	3/3 (100)	1
Recurrent hernia n/N (%)	45/421 (10.7)	2/7 (28.6)	0.173	9/37 (24.3)	0/3	1
Kingsnorth classification, n/N (%) H3 H4	345/421 (81.9) 76/421 (18.1)	5/7 (71.4) 2/7 (28.6)	0.616	28/37 (75.7) 9/37 (24.3)	1/3 (33.3) 2/3 (66.7)	0.178
Loss of domain, n/N (%)	7/41 (17.1)	1/1 (100)	0.19	3/4 (75)	0/1	0.4
PPP, n/N (%)	4/421 (1)	0/7	1	2/37 (5.4)	0/3	1
Botulinum toxin, n/N (%)	2/421 (0.5)	0/7	1	2/37 (5.4)	0/3	1
Ambulatory surgery, n/N (%)	236/421 (56.1)	4/7 (57.1)	1	18/37 (48.6)	1/3	1
Anaesthesia, n/N (%) Regional General	166/417 (39.8) 251/417 (60.2)	2/7 (28.6) 5/7 (71.4)	0.708	12/36 (33.3) 24/36 (66.7)	1/3 (33.3) 2/3 (66.7)	1
Antibiotic prophylaxis n/N (%)	324/421 (77)	6/7 (85.7)	1	33/37 (89.2)	3/3 (100)	1
Approach n/N (%) Open Laparoscopy	352/417 (84.4) 65/417 (15.6)	5/6 (83.3) 1/6 (16.7)	1	28/35 (80) 7/35 (20)	3/3 (100) 0/3	1
Conversion (n/N (%))	4/70 (5.7)	0/1	1	6/6 (100)	ND	NC
Abdominal wall experienced surgeon n/N (%)	229/421 (54.4)	4/7 (57.1)	1	27/37 (73)	0/3	0.029
CDC classification, n/N (%) I-II III-IV	420/421 (99.8) 1/421 (0.2)	7/7 (100) 0	1	37/37 (100) ND	3/3 (100) ND	NC
Mesh type n/N (%) Synthetic permanent Synthetic absorbable	419/421 (99.5) 2/421 (0.5)	7/7 (100) 0	1	36/37 (97.3) 1/37 (2.7)	3/3 (100) 0	1
Mesh position n/N (%) Anterior Posterior	302/408 (74) 106/408 (26)	5/7 (71.4) 2/7 (28.6)	1	23/37 (62.2) 14/37 (37.8)	2/2 (100) 0	0.528
Intraoperative complications n/N (%)	14/421 (3.3)	1/7 (14.3)	0.222	2/37 (5.4)	0/3	1.000
Surgical time (min) [median, (P25-P75)]	60 (45–77)	75 (52.5–90)	0.413	70 (50–90)	61.67 (20.21)	0.757
Length of hospital stay (days) [median, (P25-P75)]	1 (1–1)	2.5 (1–7)	0.007	1 (1–1)	1*	0.656
Postoperative complications at 30 POD	27/421 (6.41)	2/7 (28.5)	0.075	4/37 (10.81)	1/3 (33.3)	0.338
Follow-up (months) [mean, (SD)]	0.9 (0.22)	4.1 (6.1)	0.036	10.6 (9.29)	16 (6.93)	0.293

POD, postoperative days; N, number of patients with available information; SD, standard deviation; P25-P75, percentile 25 – percentile 75; Min, minutes; BMI, body mass index; COPD, chronic obstructive pulmonary disease; ASA, American Society of Anaesthesiologists; PPP, preoperative progressive pneumoperitoneum; CDC, US Centres for Disease Control and Prevention classification; ND, no data; NC, not calculable. *Data available for only one patient.

At maximum follow-up, recurrence was documented in three of 40 patients (7.5%) and the only factor associated with a lower risk of recurrence was the presence of an AWS-experienced surgeon (0% vs. 21.4%, P = 0.034). Given the limited number of recurrence events, analyses of 30-day recurrence and recurrence at latest available follow-up were considered exploratory and were not subjected to multivariable modelling.

## Discussion

The analysis of the EVEREG registry showed high rates of postoperative complications and SSO in inguinoscrotal repairs, which appear to exceed those generally reported in the literature for conventional inguinal hernia repairs [14–18]. Comorbidities, H4-type hernias, and longer operative time were identified as adverse prognostic factors for complications, whereas ambulatory surgery and the involvement of an AWS-experienced surgeon were associated with improved outcomes. Recurrence was infrequently reported, and the only factor linked to better results was the participation of an AWS-experienced surgeon.

Our study shows results consistent with those reported in registries [17], although most of the available literature is based on case reports and cohort studies [2, 19, 20] which include heterogeneous techniques and results. The heterogeneity in surgical management observed in the EVEREG registry reflects variation in current practice but also limits direct comparison between techniques (e.g., patient selection for open or minimally invasive repair).

Interestingly, impaired outcomes related to patient comorbidities such as age and ASA grade are also in line with current evidence [17, 21, 22]. Although these factors are largely non-modifiable and mainly reflect patient comorbidity rather than specific characteristics of the inguinoscrotal hernia, they may be useful for preoperative risk assessment.

On the other hand, scrotal hernias have been associated with higher rates of complications and SSO, such as bleeding and seroma [23, 24], as well as recurrence [2, 25]. Those results are consistent with our findings. The lower risk of complications associated with ambulatory surgery and H3-type hernias may be related to a lower complexity of these cases and patient selection. The involvement of a surgeon with experience in AWS, as suggested by the guidelines [2], was associated with significantly lower risk of complications and, despite the scarcity of data, it also appeared to have an impact on recurrence at the latest available follow-up, as previously reported [26, 27].

CDC grade and mesh type were not associated with complications, but the limited number of patients in some groups may have influenced this outcome. In contrast, operative time was related to worse outcomes and could be linked to additional procedures, repair complexity, need for conversion, or team experience and materials. Although the open approach is recommended [2] and accounted for the majority of cases, it was associated with a higher complication rate, while only a small proportion of laparoscopic procedures was performed. This finding should be interpreted very cautiously given the bordering P value (P = 0.046), the confidence interval, and the very limited number of postoperative events among patients undergoing laparoscopy and a possible selection bias (e.g., preferential use of open surgery for more complex or irreducible hernias).

However, higher conversion rates from minimally invasive to open surgery were observed compared with conventional groin hernia repairs from other series [28, 29]. Future investigations should address the role of robotic platforms in complex groin hernias and evaluate if they can improve minimally invasive surgery rates [30–32]. Similarly, open preperitoneal approaches have shown remarkable results and, although they accounted for only a small proportion (16%) of the open procedures in our study, they may represent an economical and valid alternative to minimally invasive approaches and the Lichtenstein technique [33–35]. In fact, centres with experience in this technique have reported good short- and long-term outcomes in groin hernia repair, including patients with inguinoscrotal hernias [36].

In contrast to the literature [17], postoperative complications did not appear to influence recurrence rates at the latest available follow-up; however, the limited number of recurrence cases reported may have affected this outcome and should be carefully interpreted. Additionally, recurrences during the first postoperative month may be related to technical defects or hernia persistency and should be more appropriately interpreted as an early technical failure. Nonetheless, recurrence rate at the latest available follow-up was higher than in conventional repairs [2], and the influence of an experienced surgeon might suggest that, in line with current recommendations, referral to or management in collaboration with an experienced abdominal wall surgeon should be considered in complex cases.

Limitations of our study include those inherent to registries [37, 38] and their retrospective nature, especially in terms of data incompleteness (relevant in variables such as recurrence or loss of domain) and loss to follow-up, which could strongly influence outcomes such as seroma rate or recurrence at the latest available follow-up. In fact, follow-up completion decreased markedly over time, preventing reliable estimates of recurrence at fixed 1- and 2-year intervals and limiting interpretation beyond the early postoperative period. Furthermore, participation in EVEREG is voluntary. Participating centres and surgeons may have a particular interest in AWS, and registered patients may therefore not fully represent all inguinoscrotal hernia repairs performed in Spain, so selection and reporting bias cannot be excluded. Additionally, the small proportion of H4-type hernias and cases with loss of domain makes it difficult to generalise our results to giant scrotal hernias. Moreover, the classification used was not the one recommended in the guideline [2], as the registry was initiated prior to its publication. Intraoperative complications were rarely recorded, and additional procedures such as intestinal resections or orchiectomies associated with the repair may be underreported. In addition, although postoperative complications and their Clavien-Dindo grade were recorded, the specific treatment of each complication was not systematically available in the registry. Similarly, no information was reported on the management of the sac, incidence of testicular atrophy, scrotoplasty, drainage placement, preoperative symptoms, or chronic pain, or costs. It is unknown whether patients received prehabilitation strategies to optimise baseline comorbidities or if a shared decision-making model was used, and the definition of “surgeon with experience” in AWS within the registry does not imply a formal certification. We could also not directly compare outcomes from different surgical techniques or patient selection for each. Patients who opted for watch-and-wait strategies [2, 39] were not evaluated. Finally, it is not possible to generalise our findings to other healthcare systems, emergency settings, or low-income countries.

To conclude, this multicentre registry analysis describes the management and short-term outcomes of elective inguinoscrotal hernia repair in participating EVEREG centres. Open repair with anterior mesh placement was the most frequently used approach, while postoperative complications occurred in approximately 7% of patients and were mostly low-grade. Patient comorbidity and greater hernia complexity were associated with worse outcomes, while surgery performed by an AWS-experienced surgeon was independently associated with fewer postoperative complications. These findings support individualized surgical planning and consideration of referral of complex cases to experienced abdominal wall teams. The available data do not allow conclusions regarding the superiority of a specific technique or long-term recurrence.

## Data Availability

The raw data supporting the conclusions of this article are available from the corresponding author upon reasonable request.
